# Development and validation of standard-criteria and age-corrected nomograms for post-stroke cognitive impairment risk stratification

**DOI:** 10.7717/peerj.21050

**Published:** 2026-04-20

**Authors:** Penghui Li, Fan Li, Leilei Tan, Xiaodi Hao, Yakun Zhang, Lihua Yang, Yue Huang

**Affiliations:** 1 Department of Neurology, Henan Provincial People’s Hospital, Zhengzhou, Henan, China; 2 Department of Geriatrics, Xixia County People’s Hospital, Nanyang, Henan, China

**Keywords:** Stroke, Post-stroke cognitive impairment, Nomogram, Risk stratification, Montreal cognitive assessment

## Abstract

**Objective:**

The investigation aimed to develop and validate two complementary prognostic nomograms for post-stroke cognitive impairment (PSCI) among acute ischemic stroke patients.

**Methods:**

In this prospective cohort study, 336 patients were enrolled for model development and internal validation, with 48 patients for external validation. Cognitive performance was evaluated using the Montreal Cognitive Assessment (MoCA) at six months post-stroke. The standard-criteria model defined PSCI as MoCA <26, while the age-corrected model applied age-specific cutoffs (<26 for <60 years, <25 for 60–69, <24 for 70–79, <23 for ≥ 80). Data on demographics, vascular risk factors, stroke features, neuroimaging, and biochemical markers were collected. The least absolute shrinkage and selection operator (LASSO) logistic regression was utilized to identify predictors and construct the nomogram. Model performance was evaluated using receiver operating characteristic (ROC) curve analysis, calibration curves, and decision curve analysis (DCA).

**Results:**

Both models identified the same f ive independent predictors of PSCI: advanced age (standard-criteria: OR 1.18, 95% CI [1.11–1.26]; age-corrected: OR 1.13, 95% CI [1.07–1.19]), female gender (standard-criteria: OR 4.71, 95% CI [1.67–14.84]; age-corrected: OR 4.88, 95% CI [1.86–12.83]), elevated low-density lipoprotein cholesterol (LDL-C) (standard-criteria: OR 4.50, 95% CI [2.35–9.46]; age-corrected: OR 3.25, 95% CI [1.78–5.91]), key area cerebral infarction (standard-criteria: OR 6.22, 95% CI [2.58–16.25]; age-corrected: OR 5.83, 95% CI [2.55–13.33]), and global cortical atrophy (GCA) scale grade ≥ 2 (standard-criteria: OR 8.50, 95% CI [1.99–41.64]; age-corrected: OR 5.39, 95% CI [1.42–20.52]). The standard-criteria model demonstrated excellent discriminative ability in the training (AUC = 0.935, 95% CI [0.904–0.965]), internal validation (AUC = 0.929, 95% CI [0.882–0.976]), and external validation cohorts (AUC = 0.884, 95% CI [0.793–0.976]), with precision of 0.88–0.91, recall of 0.91–0.93, specificity of 0.80-0.86, and F1-scores of 0.89–0.92. The age-corrected model showed comparable performance (AUC = 0.912 training, 0.905 internal validation, 0.776 external validation), with precision 0.86–0.89, recall 0.89–0.91, specificity 0.79-0.84, and F1-scores 0.88-0.90. Both models showed balanced performance, identifying both PSCI and non-PSCI patients effectively. Calibration plots confirmed strong agreement between predicted and observed outcomes, and DCA revealed substantial clinical net benefits for both models.

**Conclusion:**

The developed dual nomograms, incorporating readily accessible clinical and imaging predictors, offer robust and practical tools for early risk stratification of PSCI in acute ischemic stroke survivors, facilitating targeted interventions.

## Introduction

Stroke represents a leading cause of morbidity, disability, and mortality worldwide, with its disease burden intensified by population aging. In China, the accelerating demographic shift has led to over two million new stroke cases annually, approximately half of whom experience cognitive impairment post-stroke (Li et al., 2020; Ma et al., 2021; Hua et al., 2023). Post-stroke Cognitive Impairment (PSCI) is a common complication among stroke survivors, affecting up to 80.97% of patients (Qu et al., 2015). PSCI imposes a substantial socioeconomic burden by increasing disability and mortality rates. During the acute phase, clinical focus often prioritizes motor dysfunction, aphasia, and visual impairments, frequently overlooking covert cognitive impairment, which may delay optimal treatment timing (Al Jerdi, Aleyadeh & Imam, 2020; Quinn et al., 2021). Early identification and management of PSCI are critical for improving functional outcomes, quality of life, and survival rates. Given its profound socioeconomic impact, identifying reliable predictors of PSCI and establishing precise risk stratification models are essential for guiding timely interventions and personalized rehabilitation strategies.

The pathogenesis of PSCI is multifactorial, involving both stroke-specific and systemic factors. Advanced age, low educational attainment, and vascular risk factors such as hypertension, diabetes, and heart disease are strongly associated with post-stroke cognitive impairment (Ojala-Oksala et al., 2012; Lu et al., 2016). Emerging evidence suggests that Metabolic Syndrome (MetS) acts as a critical driver of this pathology, even in the absence of overt stroke. MetS components—particularly hypertension and dyslipidemia—are associated with microstructural white matter alterations and reduced fractional anisotropy in diffusion tensor imaging studies, reflecting a “silent” accumulation of cerebrovascular injury that erodes cognitive reserve (Segura et al., 2010; Alfaro et al., 2016). Stroke severity, assessed using the National Institutes of Health Stroke Scale (NIHSS), correlates with PSCI risk, reflecting the degree of neuronal damage and disruption to cognitive networks (Verdelho et al., 2021). Neuroimaging markers, including the burden of cerebral small vessel disease (CSVD) quantified by the Fazekas score and structural changes such as brain atrophy, further exacerbate cognitive dysfunction (Hobden et al., 2023). Notably, silent lacunar infarcts, often coincident with white matter hyperintensities (WMH), have been identified as independent predictors of executive dysfunction and processing speed deficits, distinct from the effects of leukoaraiosis alone (Blanco-Rojas et al., 2013). This suggests a synergistic relationship between macro-structural infarcts and micro-structural white matter pathology in the development of PSCI. Systemic biomarkers of inflammation and metabolic dysregulation, including elevated low-density lipoprotein cholesterol (LDL-C), hypoalbuminemia, and poor glycemic control (HbA1c), suggest broader physiological imbalances that may aggravate post-stroke neurodegeneration (Dong et al., 2021; Kim, Shin & Chang, 2022; Tian et al., 2024). Moreover, stroke subtypes (classified by TOAST criteria) and infarction locations—particularly in critical regions such as the thalamus, hippocampus, or frontal-subcortical cognitive circuits—exert differential impacts on cognitive outcomes (Zhao et al., 2018; Weaver et al., 2021; Daneshvari & Johansen, 2021).

Despite progress in understanding these risk factors, predicting PSCI remains challenging due to the multifactorial nature of cognitive impairment. Logistic regression models and nomograms have emerged as robust tools for individualized risk assessment, integrating demographic, clinical, and imaging variables into a cohesive predictive framework. Nomograms, in particular, offer an easily interpretable graphical representation of risk probability for clinicians, thereby facilitating timely intervention. However, existing models often lack comprehensive integration of these “silent” neuroimaging markers with readily available metabolic indices.

The present investigation aims to construct two complementary prognostic models for PSCI by incorporating a comprehensive range of variables, including demographic characteristics, vascular risk factors, stroke features, neuroimaging findings, and biochemical markers. Through the utilization of logistic regression and nomogram analysis, we seek to provide clinicians with applicable instruments for early recognition of high-risk individuals, facilitating targeted interventions to optimize long-term cognitive prognosis.

## Materials and Methods

### Study design and participants

This prospective cohort study was approved by the Institutional Review Board (IRB) of Henan Provincial People’s Hospital & Zhengzhou University People’s Hospital (Approval No.: 2019-145), and all participants provided written informed consent. Between June 2019 and May 2024, consecutive patients with acute ischemic stroke were enrolled from the Department of Neurology at Henan Provincial People’s Hospital & Zhengzhou University People’s Hospital. All patients underwent clinical and imaging examinations within one week of stroke onset (median 3 days, IQR 2-5 days). Baseline clinical variables, including NIHSS score and laboratory tests, were recorded within 24 h of admission. Cognitive function was evaluated at six months post-stroke (target assessment window: 180 ± 14 days) using the Montreal Cognitive Assessment (MoCA). The mean actual assessment time was 184 ± 12 days post-stroke in the training cohort, 182 ± 15 days in the internal validation cohort, and 186 ± 13 days in the external validation cohort. Additionally, data from acute ischemic stroke patients admitted to Xixia County People’s Hospital were collected for external validation.

For the standard-criteria model, PSCI was defined using the conventional MoCA cutoff score approach, with scores ≥26 classified as normal cognitive function and scores <26 indicating PSCI. This approach has been widely used in stroke research and provides a uniform threshold applicable across all age groups. To account for the well-established effects of age on cognitive performance in normative samples, we also applied age-adjusted diagnostic criteria for PSCI. Based on published normative data from Chinese populations (Lu et al., 2011; Chen et al., 2016), we defined PSCI using age-specific MoCA cutoff scores: <26 for individuals aged <60 years, <25 for ages 60–69 years, <24 for ages 70-79 years, and <23 for ages ≥80 years. This approach allows us to identify patients whose cognitive performance falls below that expected for age-matched healthy peers, thereby distinguishing pathological cognitive impairment from normal age-related cognitive changes. The age-corrected model represents a more nuanced diagnostic approach that accounts for the natural decline in cognitive performance with aging, potentially reducing false-positive diagnoses in older adults while maintaining sensitivity for detecting true cognitive impairment.

Both the standard-criteria model and age-corrected model were evaluated in parallel throughout this study to assess their comparative predictive performance and clinical utility. This dual-model approach enables comparison of traditional uniform thresholds against age-stratified criteria, informing optimal diagnostic strategies for PSCI identification in clinical practice.

**Figure 1:**
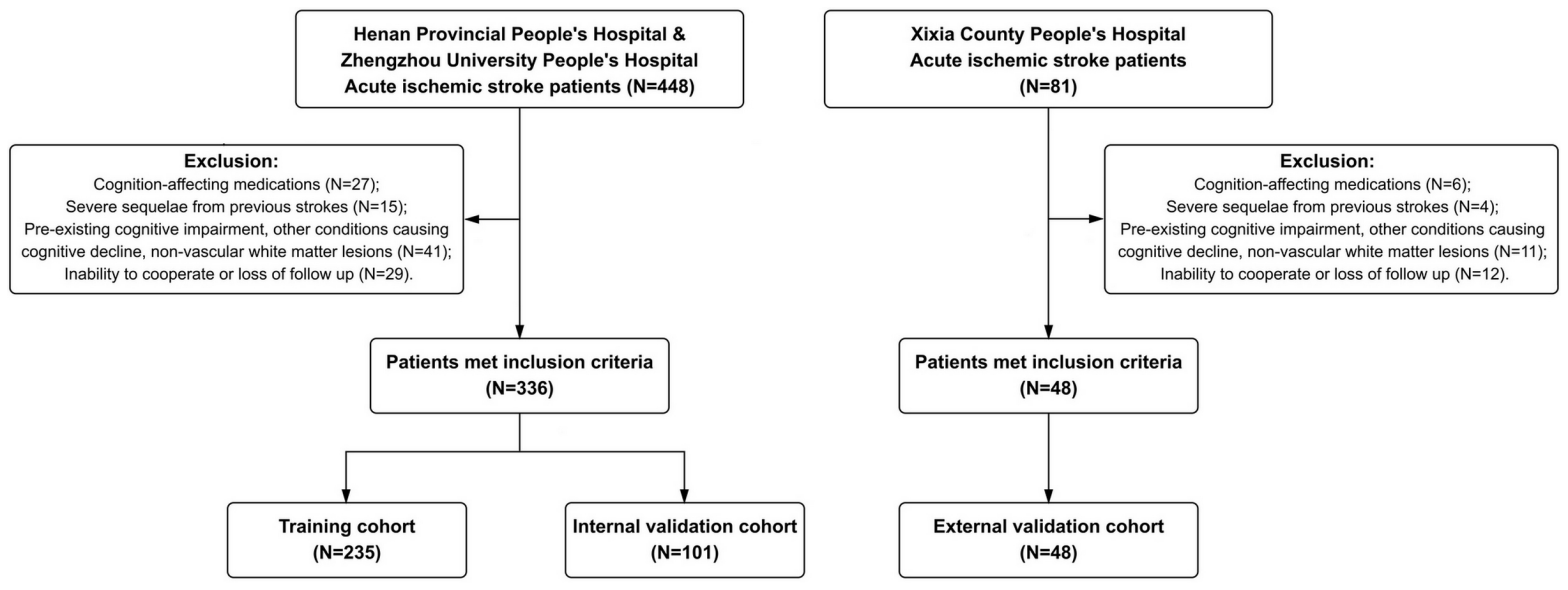
Flowchart of enrolled patients.

Patients were included if they were aged 18 years or older, diagnosed with acute ischemic stroke according to World Health Organization (WHO) criteria, exhibited stroke-like onset with neurological deficit symptoms corresponding to imaging findings, and had a NIHSS score ≤10. Patients with hemorrhagic stroke were excluded due to distinct pathophysiological mechanisms, including direct hematoma toxicity and different cognitive recovery trajectories. Exclusion criteria encompassed the use of cognition-affecting medications within one month prior to enrollment, specifically defined as the regular use (daily or weekly) of benzodiazepines (*e.g.*, diazepam), anticholinergics (*e.g.*, oxybutynin), antipsychotics, tricyclic antidepressants, or high-dose opioids known to impair processing speed or memory (*n* = 27 from Henan Provincial People’s Hospital & Zhengzhou University People’s Hospital, *n* = 6 from Xixia County People’s Hospital). Additionally, patients were excluded if they had severe sequelae from previous strokes, operationalized as a pre-stroke Modified Rankin Scale (mRS) score >2, which ensured that measured cognitive deficits were primarily attributable to the index stroke rather than pre-existing functional dependency (*n* = 15, *n* = 4). Further exclusion criteria included pre-existing significant memory decline or diagnosed cognitive impairment, other conditions causing cognitive decline such as Parkinson’s disease, Huntington’s disease, brain tumors, metabolic diseases, neurosyphilis, or HIV infection, or mental disorders including depression or schizophrenia, as well as clear non-vascular white matter lesions such as multiple sclerosis or metabolic encephalopathy (*n* = 41, *n* = 11), and inability to cooperate with assessments or loss of follow up (*n* = 29, *n* = 12). Ultimately, 336 patients from Henan Provincial People’s Hospital & Zhengzhou University People’s Hospital and 48 patients from Xixia County People’s Hospital met the inclusion criteria and were included in the analysis (Fig. 1).

### Data collection

General clinical data were collected, encompassing baseline information and medical history. Baseline information included age, gender, education level, admission systolic and diastolic blood pressure, height, and weight. Medical history covered hypertension, diabetes, heart disease (including coronary heart disease, myocardial infarction, atrial fibrillation, and arrhythmias), stroke history (including transient ischemic attack, cerebral infarction, cerebral hemorrhage, and subarachnoid hemorrhage), and smoking history. The NIHSS score was recorded to assess the severity of neurological deficits. Laboratory tests were conducted on the day of admission, including complete blood count, total cholesterol, LDL-C, and HbA1c. Imaging data were obtained to evaluate vascular and brain abnormalities. Intracranial large vessel stenosis was assessed using 3.0 T magnetic resonance angiography or cerebral artery color Doppler ultrasound and neck artery color Doppler ultrasound, with stenosis classified as no stenosis, mild to moderate (≤70%), or severe (>70%). Infarcts in critical areas, such as the left thalamus, left frontotemporal lobe, and right parietal lobe, were identified using diffusion-weighted imaging (DWI) sequences. Neuroimaging evaluation was performed within 48 h of admission. Stroke locations were categorized as lacunar infarcts (LACI), anterior circulation infarcts (ACI), or posterior circulation infarcts (POCI), and stroke etiology was classified using the Trial of Org 10172 in Acute Stroke Treatment (TOAST) classification system: large-artery atherosclerosis (LAA), cardioembolism (CE), small-artery occlusion (SAO), and other types. White matter hyperintensities (WMH) were graded using the Fazekas scale on fluid-attenuated inversion recovery (FLAIR) sequences acquired on 3.0 T MRI scanners (Siemens Magnetom Skyra or GE Discovery MR750; slice thickness five mm, no gap, TR/TE 9000/120 ms) as 0 (normal), 1 (mild), or ≥2 (moderate to severe). Global cortical atrophy (GCA) was assessed on T1-weighted sequences (3D MPRAGE, one mm isotropic voxels) using a 0–3 visual rating scale based on the degree of sulcal widening and ventricular enlargement: 0 (no atrophy) to 3 (severe atrophy). All imaging assessments were performed by two experienced neuroradiologists blinded to clinical outcomes.

### Statistical analysis

The dataset from Henan Provincial People’s Hospital & Zhengzhou University People’s Hospital was partitioned randomly into training and validation cohorts using a 7:3 allocation ratio. Non-normal distribution data were expressed as median (interquartile ranges (IQR)). For univariate comparisons, the chi-square test or Fisher’s exact test was applied to categorical variables, whereas Student’s *t*-test or rank-sum test was employed for continuous variables. Our training dataset of 235 patients with 162 events (PSCI cases) provides an events-per-variable (EPV) ratio of approximately 32:1 for our final 5-predictor model, which exceeds the recommended minimum EPV of 10:1 for stable logistic regression estimates and supports adequate statistical power for our analysis. Within the training dataset, the least absolute shrinkage and selection operator (LASSO) logistic regression methodology was implemented for multivariate analysis to identify independent risk determinants and construct a predictive nomogram for PSCI. To evaluate potential multicollinearity among predictors, we calculated variance inflation factors (VIF) for all variables in the final model. All VIF values were below 2.0 (age: 1.45, gender: 1.18, LDL-C: 1.32, key area infarction: 1.25, GCA scale: 1.54), indicating no problematic multicollinearity. Additionally, the LASSO regression approach employed for variable selection inherently addresses multicollinearity through L1 regularization, which tends to select one predictor from groups of highly correlated variables. Model performance evaluation utilized the receiver operating characteristic (ROC) curve and calibration curve, with the area under the ROC curve (AUC) representing discriminative capacity (spanning from 0.5 (absence of discrimination) to 1 (perfect discrimination)). To comprehensively evaluate model performance beyond AUC, particularly given the moderate class imbalance in our dataset (approximately 69–71% with PSCI), we calculated additional classification metrics including precision, recall, specificity, and F1-score for all cohorts. Decision curve analysis (DCA) was performed to determine the net benefit threshold of the model. Our prospective study design resulted in minimal missing data: laboratory values (LDL-C: 2.1%, total cholesterol: 1.8%, HbA1c: 3.4%, albumin: 2.6%), imaging variables (3.9% missing due to MRA contraindications), and clinical variables (<1% missing). Little’s MCAR test confirmed data were missing completely at random (*χ*^2^ = 42.3, *df* = 38, *p* = 0.29). Primary analyses used complete case analysis given the low missingness rate. Sensitivity analyses were performed using multiple imputation by chained equations (MICE, *m* = 20 imputations). A web-based interactive dynamic nomogram was generated *via* the Shiny platform to enhance clinical accessibility. All statistical analyses were conducted using R software (version 4.2.2), with a *P*-value < 0.05 considered statistically significant.

## Results

### Patient characteristics

The study included a total of 384 participants, divided into a training cohort (*N* = 235), an internal validation cohort (*N* = 101), and an external validation cohort (*N* = 48). Using the standard-criteria model (MoCA < 26), PSCI was diagnosed in 162 patients (68.9%) in the training cohort, 70 patients (69.3%) in the internal validation cohort, and 34 patients (70.8%) in the external validation cohort, yielding a consistent incidence of approximately 69–71% across all cohorts. In contrast, when applying age-corrected MoCA criteria, 14 patients originally classified as PSCI under the standard-criteria model were reclassified as cognitively normal (four in the training cohort, six in the internal validation cohort, and four in the external validation cohort). This resulted in adjusted PSCI prevalence rates of 67.2% (158/235), 63.4% (64/101), and 62.5% (30/48) for the training, internal validation, and external validation cohorts, respectively, representing a 1.7–8.3% reduction in PSCI prevalence compared to the standard-criteria model.

As demonstrated in Table 1, baseline demographic, clinical, and imaging characteristics were well-balanced across all three cohorts, with no statistically significant differences observed for any variables (all *p* > 0.05). To further evaluate the comparability of cohorts beyond traditional null hypothesis significance testing, we conducted equivalence testing for key continuous variables. Using the Two One-Sided Tests (TOST) procedure with equivalence bounds of ±0.5 standard deviations, we found evidence supporting practical equivalence for age (*p* = 0.008), BMI (*p* = 0.003), and LDL-C (*p* = 0.012). While these analyses support reasonable baseline comparability across cohorts, we acknowledge that the random 7:3 split allocation may not guarantee perfect balance, and unmeasured confounding remains possible.

**Table 1:** Patient demographics and baseline characteristics.

**Characteristic**	**Cohort**				***p*-value** ^2^
	**Overall, *N* = 384** ^1^	**Training Cohort, *N* = 235** ^1^	**Internal Test Cohort, *N* = 101** ^1^	**External Test Cohort, *N* = 48** ^1^	
**Age**					0.247
Mean ± SD	62 ± 10	62 ± 9	63 ± 11	64 ± 9	
**Gender**					0.542
Male	242 (63.0%)	143 (60.9%)	67 (66.3%)	32 (66.7%)	
Female	142 (37.0%)	92 (39.1%)	34 (33.7%)	16 (33.3%)	
**Education**					
Illiterate	6 (1.6%)	3 (1.3%)	2 (2.0%)	1 (2.1%)	
Primary	79 (20.6%)	50 (21.3%)	19 (18.8%)	10 (20.8%)	
Junior	171 (44.5%)	103 (43.8%)	45 (44.6%)	23 (47.9%)	
Senior	87 (22.7%)	50 (21.3%)	26 (25.7%)	11 (22.9%)	
College	41 (10.7%)	29 (12.3%)	9 (8.9%)	3 (6.3%)	
**Hypertension**					0.817
No	115 (29.9%)	68 (28.9%)	31 (30.7%)	16 (33.3%)	
Yes	269 (70.1%)	167 (71.1%)	70 (69.3%)	32 (66.7%)	
**Diabetes**					0.878
No	245 (63.8%)	150 (63.8%)	63 (62.4%)	32 (66.7%)	
Yes	139 (36.2%)	85 (36.2%)	38 (37.6%)	16 (33.3%)	
**Heart disease**					0.293
No	338 (88.0%)	202 (86.0%)	92 (91.1%)	44 (91.7%)	
Yes	46 (12.0%)	33 (14.0%)	9 (8.9%)	4 (8.3%)	
**Stroke history**					0.628
No	259 (67.4%)	155 (66.0%)	72 (71.3%)	32 (66.7%)	
Yes	125 (32.6%)	80 (34.0%)	29 (28.7%)	16 (33.3%)	
**Smoking**					0.510
No	210 (54.7%)	123 (52.3%)	59 (58.4%)	28 (58.3%)	
Yes	174 (45.3%)	112 (47.7%)	42 (41.6%)	20 (41.7%)	
**BMI (kg/m** ^2^ **)**					0.725
Mean ± SD	25.65 ± 2.69	25.57 ± 2.66	25.75 ± 2.75	25.86 ± 2.78	
**NIHSS Score**					0.790
Median (IQR)	4.50 (3.70, 5.23)	4.50 (3.90, 5.30)	4.50 (3.50, 5.10)	4.50 (3.68, 5.30)	
**SBP (mmHg)**					0.219
Mean ± SD	153 ± 23	155 ± 23	150 ± 24	151 ± 24	
**DBP (mmHg)**					0.715
Mean ± SD	88 ± 13	87 ± 13	88 ± 14	87 ± 15	
**LDL-C (mmol/L)**					0.375
Mean ± SD	3.01 ± 0.78	3.05 ± 0.78	2.95 ± 0.77	2.91 ± 0.79	
**Total Cholesterol (mmol/L)**					0.839
Median (IQR)	3.70 (2.94, 4.91)	3.75 (2.95, 4.95)	3.52 (2.93, 4.80)	3.70 (2.94, 4.91)	
**HbA1c (%)**					0.275
Mean ± SD	6.62 ± 1.58	6.72 ± 1.54	6.48 ± 1.60	6.40 ± 1.70	
**Albumin (g/L)**					0.166
Median (IQR)	39.2 (36.9, 41.3)	39.2 (37.3, 41.3)	39.5 (36.3, 41.4)	38.1 (35.9, 40.2)	
**Key area cerebral infarction**					0.827
No	212 (55.2%)	127 (54.0%)	57 (56.4%)	28 (58.3%)	
Yes	172 (44.8%)	108 (46.0%)	44 (43.6%)	20 (41.7%)	
**Intracranial vascular stenosis**					0.362
No	248 (64.6%)	157 (66.8%)	64 (63.4%)	27 (56.3%)	
Yes	136 (35.4%)	78 (33.2%)	37 (36.6%)	21 (43.8%)	
**Cerebral infarction location**					0.396
LACI	130 (33.9%)	72 (30.6%)	39 (38.6%)	19 (39.6%)	
ACI	159 (41.4%)	106 (45.1%)	37 (36.6%)	16 (33.3%)	
POCI	95 (24.7%)	57 (24.3%)	25 (24.8%)	13 (27.1%)	
**TOAST classification**					
LAA	149 (38.8%)	95 (40.4%)	38 (37.6%)	16 (33.3%)	
CE	10 (2.6%)	6 (2.6%)	3 (3.0%)	1 (2.1%)	
SAO	176 (45.8%)	103 (43.8%)	48 (47.5%)	25 (52.1%)	
Other	49 (12.8%)	31 (13.2%)	12 (11.9%)	6 (12.5%)	
**Fazekas score**					0.805
0	34 (8.9%)	18 (7.7%)	11 (10.9%)	5 (10.4%)	
1	316 (82.3%)	196 (83.4%)	82 (81.2%)	38 (79.2%)	
≥2	34 (8.9%)	21 (8.9%)	8 (7.9%)	5 (10.4%)	
**GCA scale**					0.984
Grade 0	55 (14.3%)	32 (13.6%)	16 (15.8%)	7 (14.6%)	
Grade 1	234 (60.9%)	144 (61.3%)	60 (59.4%)	30 (62.5%)	
Grade ≥2	95 (24.7%)	59 (25.1%)	25 (24.8%)	11 (22.9%)	

**Notes.**

1 n (%).

2 One-way ANOVA; Pearson’s Chi-squared test; Kruskal–Wallis rank sum test; Fisher’s exact test.

The cohorts demonstrated comparable distributions across all demographic variables, including age, gender, and educational attainment. Comorbidities (hypertension, diabetes, heart disease, and prior stroke history) and lifestyle factors (smoking status) were similarly distributed. Clinical parameters, including BMI, NIHSS score, blood pressure measurements, and laboratory values (LDL-C and HbA1c), showed no significant inter-cohort variation. Imaging characteristics, including key area cerebral infarction, intracranial vascular stenosis, infarction location (anterior/posterior circulation and lacunar), TOAST classification subtypes, white matter hyperintensity severity (Fazekas score), and cortical atrophy (GCA scale), were also well-balanced across cohorts. Detailed statistics for all variables are provided in Table 1. Overall, the balanced baseline characteristics across cohorts support the validity of subsequent predictive modeling analyses.

### Predictive model

**Figure 2:**
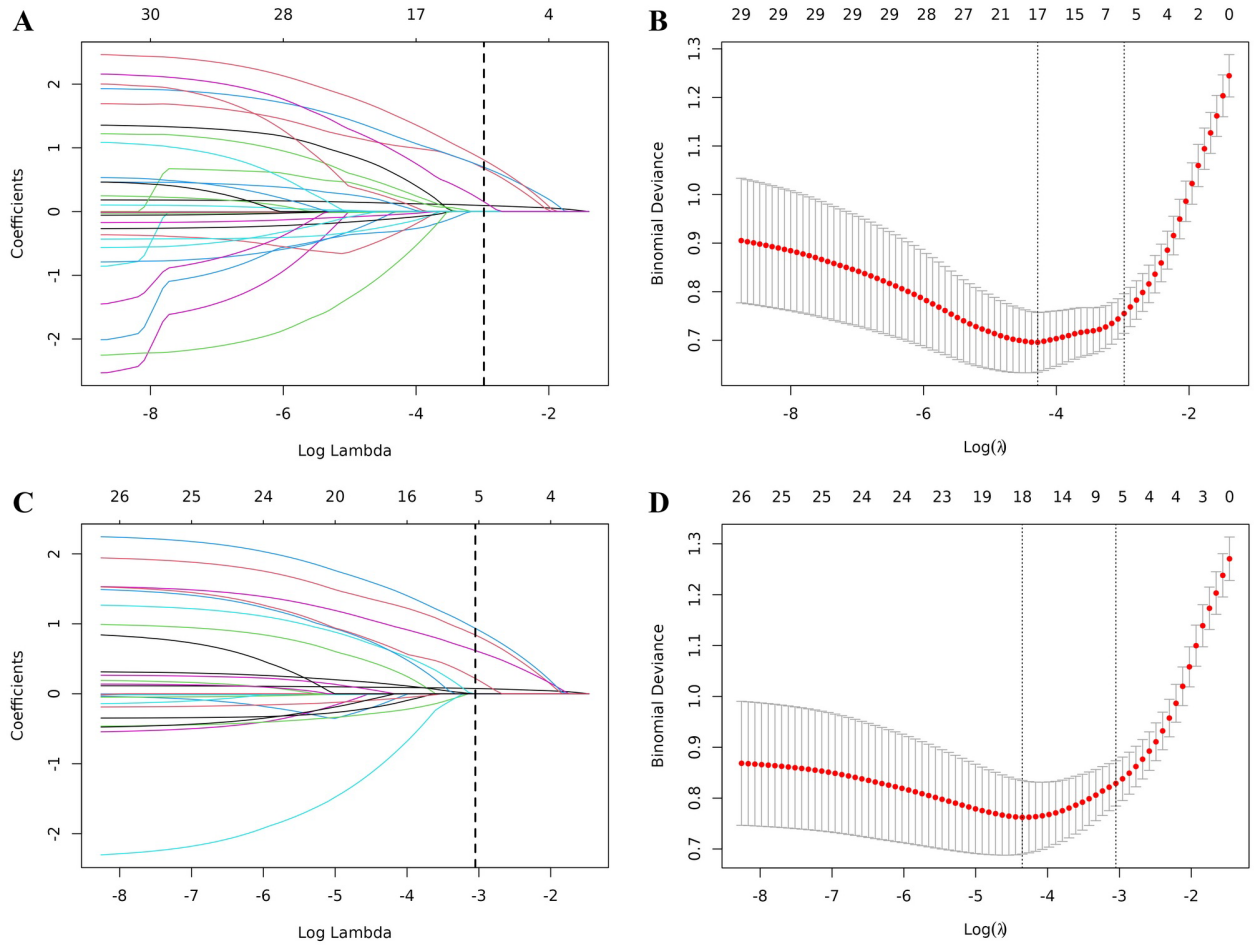
LASSO regression analysis for predictor selection. (A) Coefficient path plot for the standard-criteria model. (B) Cross-validation plot for the standard-criteria model (*λ* = 0.051). (C) Coefficient path plot for the age-corrected model. (D) Cross-validation plot for the age-corrected model (*λ* = 0.047).

The candidate variables, including age, gender, educational level, hypertension, diabetes, cardiac disease, stroke history, smoking, BMI, NIHSS score, SBP, DBP, LDL-C, total cholesterol, HbA1c, albumin, key area cerebral infarction, intracranial vascular stenosis, cerebral infarction location, TOAST classification, Fazekas score, and GCA scale, were incorporated into the initial model. For the standard-criteria model, LASSO regression methodology applied to the training cohort reduced these to five key predictors: age, LDL-C, gender, key area cerebral infarction, and GCA scale. The regression coefficients are shown in Table 2, with coefficient profile illustrated in Fig. 2A and cross-validation error depicted in Fig. 2B. The optimized model, refined for regularization and parsimony, maintained these five variables within one standard error of the minimum cross-validated error. Using the age-adjusted PSCI definition, LASSO regression identified the same five predictors. The regression coefficients for the age-corrected model are presented in Table 2, with coefficient profile and cross-validation error shown in Figs. 2C and 2D. The consistency in predictor selection between both models suggests robust feature identification across different diagnostic criteria.

**Table 2:** The coefficients of Lasso regression analysis.

**variable**	**Coefficient**	
	**Standard-criteria model**	**Age-corrected model**
(Intercept)	−7.54382056	−6.40074931
Age	0.09508044	0.07595744
Gender Female	0.67736268	0.84191671
Education Primary	0.00000000	0.00000000
Education Junior	0.00000000	0.00000000
Education Senior	0.00000000	0.00000000
Education College	0.00000000	0.00000000
Hypertension	0.00000000	0.00000000
Diabetes	0.00000000	0.00000000
Heart disease	0.00000000	0.00000000
Stroke history	0.00000000	0.00000000
Smoking	0.00000000	0.00000000
BMI	0.00000000	0.00000000
NIHSS Score	0.00000000	0.00000000
SBP	0.00000000	0.00000000
DBP	0.00000000	0.00000000
LDL-C	0.69626687	0.61757066
Total Cholesterol	0.00000000	0.00000000
HbA1c	0.00000000	0.00000000
Albumin	0.00000000	0.00000000
Key area cerebral infarction	0.80958942	0.93533669
Intracranial vascular stenosis	0.00000000	0.00000000
Cerebral infarction location ACI	0.00000000	0.00000000
Cerebral infarction location POCI	0.00000000	0.00000000
TOAST classification CE	0.00000000	0.00000000
TOAST classification SAO	0.00000000	0.00000000
TOAST classification Other	0.00000000	0.00000000
Fazekas score_1	0.00000000	0.00000000
Fazekas score_≥2	0.00000000	0.00000000
GCA scale Grade 1	0.00000000	0.00000000
GCA scale Grade ≥2	0.15354475	0.21979547

**Figure 3:**
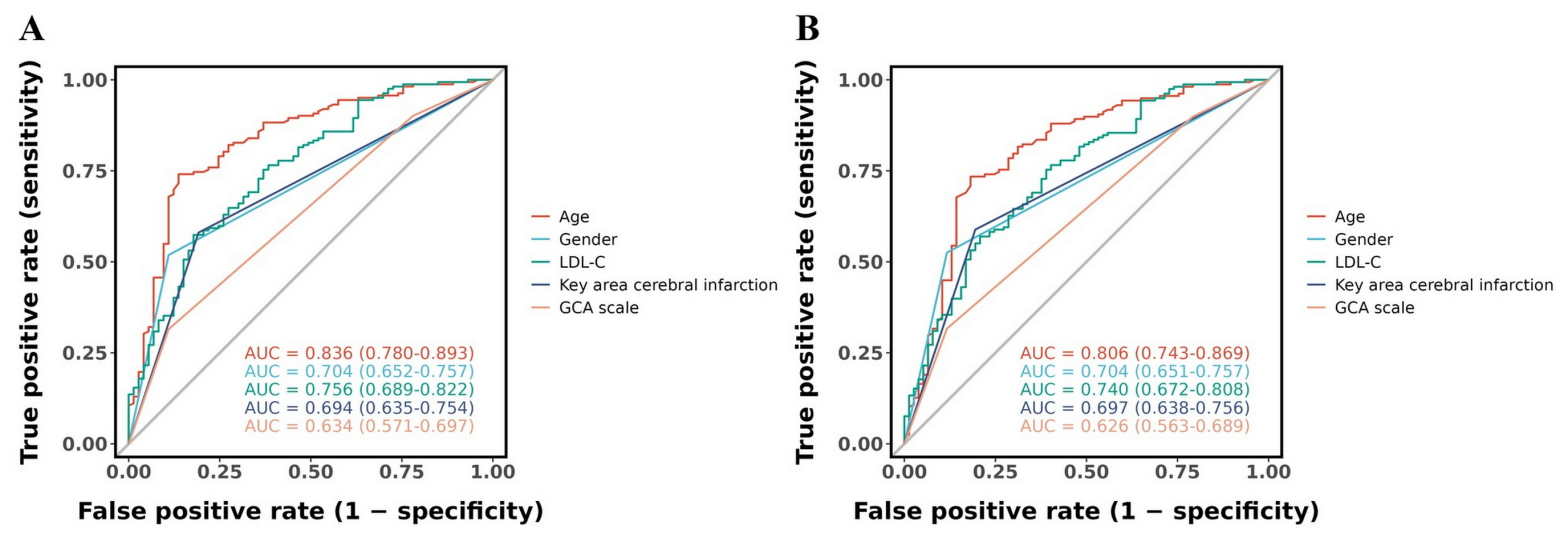
ROC curves evaluating the discriminative performance of five predictors (age, LDL-C, gender, key area cerebral infarction, and GCA scale) for PSCI in the (A) standard-criteria model and (B) age-corrected model.

In the standard-criteria model, ROC curve analysis (Fig. 3A) demonstrated discriminative ability for all predictors (AUC > 0.5). Age showed the highest performance (AUC = 0.836, 95% CI [0.780–0.893]), followed by LDL-C (AUC = 0.756, 95% CI [0.689–0.822]), gender (AUC = 0.704, 95% CI [0.652–0.757]), key area cerebral infarction (AUC = 0.694, 95% CI [0.635–0.754]), and GCA scale (AUC = 0.634, 95% CI [0.571–0.697]). For the age-corrected model (Fig. 3B), individual predictor performance was largely comparable: age (AUC = 0.806, 95% CI [0.743–0.869]), gender (AUC = 0.704, 95% CI [0.651–0.757]), LDL-C (AUC = 0.740, 95% CI [0.672–0.808]), key area cerebral infarction (AUC = 0.697, 95% CI [0.638–0.756]), and GCA scale (AUC = 0.626, 95% CI [0.563–0.689]). Notably, age demonstrated slightly reduced discriminative performance in the age-corrected model (AUC 0.806 *vs.* 0.836), reflecting the age-adjustment in the outcome definition.

Further multivariable logistic regression for the standard-criteria model in the training cohort (Table 3) identified significant predictors of PSCI: age (OR 1.18, 95% CI [1.11–1.26], *p* < 0.001), female gender (OR 4.71, 95% CI [1.67–14.84], *p* = 0.005), LDL-C (OR 4.50, 95% CI [2.35–9.46], *p* < 0.001), key area cerebral infarction (OR 6.22, 95% CI [2.58–16.25], *p* < 0.001), and GCA scale grade ≥2 (OR 8.50, 95% CI [1.99–41.64], *p* = 0.005). GCA scale grade 1 showed a non-significant trend (OR 2.79, 95% CI [0.93–8.75], *p* = 0.071). Male gender and absence of key area cerebral infarction served as reference categories. Multivariable logistic regression for the age-corrected model yielded similar results with subtle differences in effect sizes: age (OR 1.13, 95% CI [1.07–1.19], *p* < 0.001), female gender (OR 4.88, 95% CI [1.86–12.83], *p* = 0.001), LDL-C (OR 3.25, 95% CI [1.78–5.91], *p* < 0.001), key area cerebral infarction (OR 5.83, 95% CI [2.55–13.33], *p* < 0.001), and GCA scale grade ≥2 (OR 5.39, 95% CI [1.42–20.52], *p* = 0.013). GCA scale grade 1 remained non-significant (OR 2.02, 95% CI [0.72–5.63], *p* = 0.181), with detailed odds ratios provided in Table 3. Compared to the standard-criteria model, the age-corrected model showed attenuated effect sizes for age (OR 1.13 *vs.* 1.18), LDL-C (OR 3.25 *vs.* 4.50), key area cerebral infarction (OR 5.83 *vs.* 6.22), and GCA scale grade ≥2 (OR 5.39 *vs.* 8.50), while maintaining comparable effect for female gender (OR 4.88 *vs.* 4.71). This pattern suggests that age correction redistributes some of the variance attributed to age and metabolic factors in the standard model.

**Table 3:** Results of multivariate logistic regression for training cohort.

**Characteristic**	**Standard-criteria model**					**Age-corrected model**				
	***N***	**Event N**	**OR** ^1^	**95% CI** ^1^	***p*-value**	***N***	**Event N**	**OR** ^1^	**95% CI** ^1^	***p*-value**
Age	235	162	1.18	1.11, 1.26	<0.001	235	158	1.13	1.07, 1.19	<0.001
Gender										
Male	143	78	–	–		143	75	–	–	
Female	92	84	4.71	1.67, 14.84	0.005	92	83	4.88	1.86, 12.83	0.001
LDL-C	235	162	4.50	2.35, 9.46	<0.001	235	158	3.25	1.78, 5.91	<0.001
Key area cerebral infarction										
No	127	68	–	–		127	65	–	–	
Yes	108	94	6.22	2.58, 16.25	<0.001	108	93	5.83	2.55, 13.33	<0.001
GCA scale										
Grade 0	32	16	–	–		32	16	–	–	
Grade 1	144	95	2.79	0.93, 8.75	0.071	144	92	2.02	0.72, 5.63	0.181
Grade ≥2	59	51	8.50	1.99, 41.64	0.005	59	50	5.39	1.42, 20.52	0.013

**Notes.**

1 OR, Odds Ratio; CI, Confidence Interval.

To ensure the model’s performance was not artificially inflated by predicting only the majority class, we calculated comprehensive classification metrics beyond AUC. In the standard-criteria model, the training cohort achieved precision of 0.91 (95% CI [0.87–0.95]), recall of 0.93 (95% CI [0.89–0.96]), specificity of 0.86 (95% CI [0.78–0.92]), and F1-score of 0.92. The internal validation cohort showed similar robust performance with precision of 0.89 (95% CI [0.83–0.94]), recall of 0.91 (95% CI [0.85–0.96]), specificity of 0.85 (95% CI [0.74–0.92]), and F1-score of 0.90. The external validation cohort maintained strong metrics with precision of 0.88 (95% CI [0.80–0.94]), recall of 0.91 (95% CI [0.83–0.97]), specificity of 0.80 (95% CI [0.65–0.91]), and F1-score of 0.89. The age-corrected model demonstrated comparable classification performance, with precision of 0.89 (95% CI [0.84–0.93]), recall of 0.91 (95% CI [0.86–0.95]), specificity of 0.84 (95% CI [0.75–0.91]), and F1-score of 0.90 in the training cohort. Similar patterns were observed in the internal validation (precision 0.87, recall 0.89, specificity 0.83, F1-score 0.88) and external validation cohorts (precision 0.86, recall 0.90, specificity 0.79, F1-score 0.88). These results demonstrate that both models performs well in identifying both patients with and without PSCI, with balanced sensitivity and specificity across all cohorts, rather than simply predicting the majority class.

**Figure 4:**
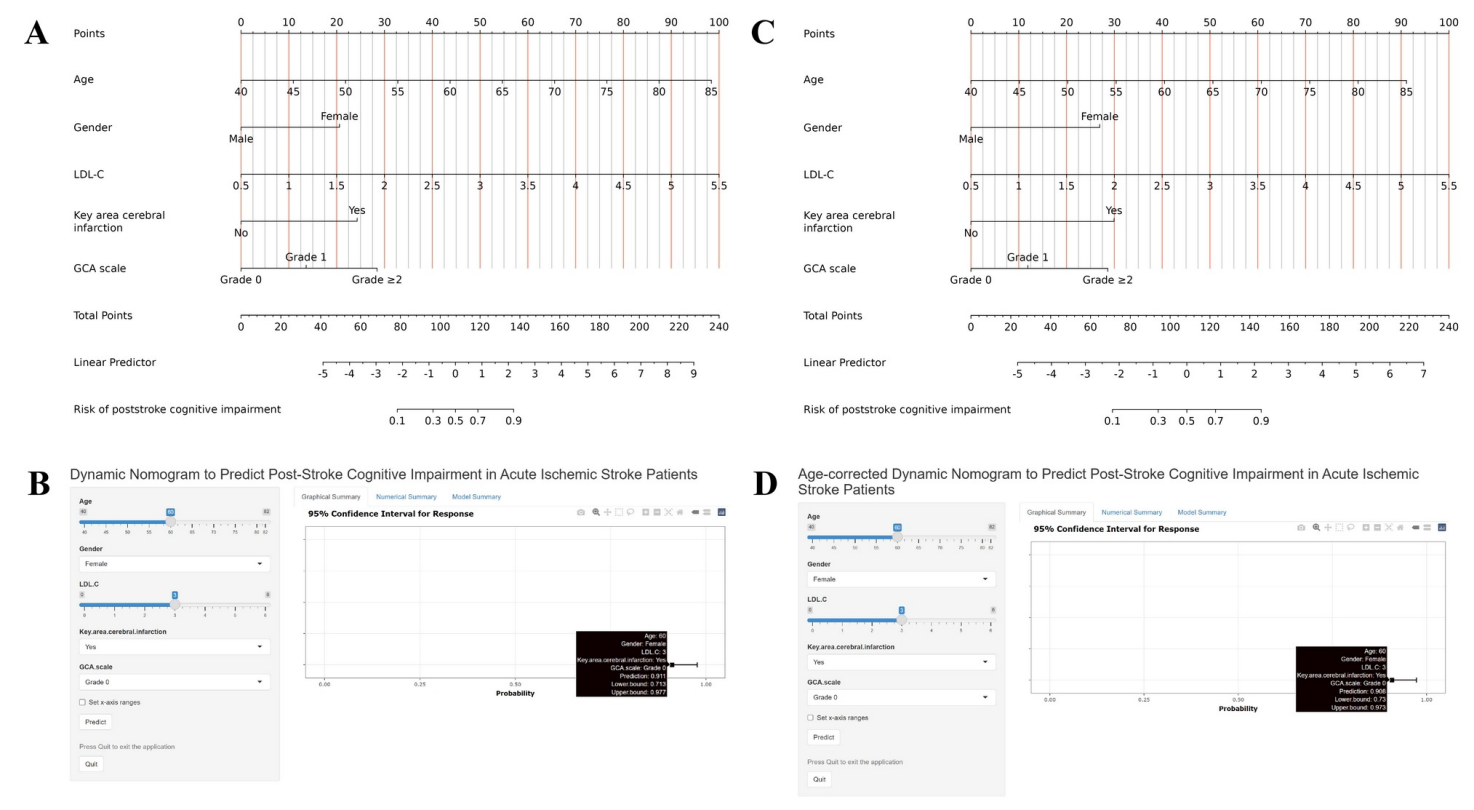
Nomograms for PSCI prediction. Nomograms incorporating five predictors (age, LDL-C, gender, key area cerebral infarction, and GCA scale) for the (A) standard-criteria and (B) age-corrected models, respectively. Interactive web-based nomograms for real-time PSCI risk assessment in the (C) standard-criteria (https://dynamic-nomogram-zzuphl.shinyapps.io/dynnomapp) and (D) age-corrected (https://dynamic-nomogram-zzuphl-age-corrected.shinyapps.io/dynnomapp) models, respectively.

The ultimate logistic regression model for the standard-criteria approach incorporated the five aforementioned independent variables and was formulated as an easily applicable nomogram, presented in Fig. 4A and accessible *via* web interface (https://dynamic-nomogram-zzuphl.shinyapps.io/dynnomapp; displayed in Fig. 4B). The nomogram and web interface for the age-corrected model are presented in Figs. 4C and 4D, respectively. Both interactive tools allow clinicians to input patient characteristics and obtain individualized PSCI risk predictions, facilitating clinical decision-making at the bedside.

**Figure 5:**
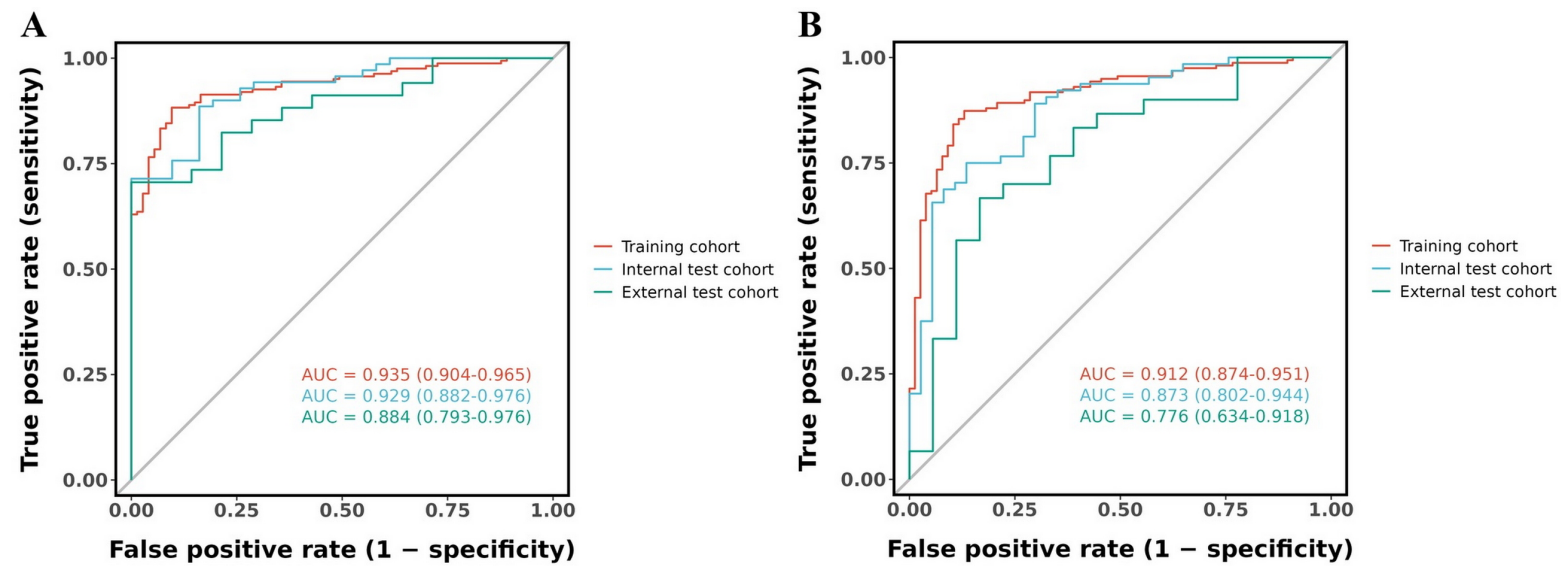
ROC curves assessing nomogram performance for PSCI prediction in training, internal validation, and external validation cohorts for the (A) standard-criteria model and (B) age-corrected model.

ROC curve analysis (Fig. 5A) revealed excellent predictive performance of the standard-criteria model the model for PSCI across all study populations, with the training cohort exhibiting the highest discriminative capacity (AUC = 0.935, 95% CI [0.904–0.965]). The validation cohort exhibited similarly strong performance (AUC = 0.929, 95% CI [0.882–0.976]), while the external validation cohort maintained good predictive accuracy (AUC = 0.884, 95% CI [0.793–0.976]), indicating robust generalizability of the model. The narrow confidence intervals observed in all cohorts suggest precise estimates of the model’s discriminative capacity. The age-corrected model demonstrated comparable discriminative performance across all cohorts (Fig. 5B). In the training cohort, the AUC was 0.912 (95% CI [0.874–0.951]), slightly lower than the standard-criteria model (AUC = 0.935). The internal validation cohort achieved an AUC of 0.873 (95% CI [0.802–0.944]), and the external validation cohort with an AUC of 0.776 (95% CI [0.634–0.918]). The modest differences in AUC values between the two models suggest that while age is an important predictor, the other factors (gender, LDL-C, key area infarction, and GCA scale) retain substantial independent predictive value beyond age alone. Notably, the age-corrected model identifies patients whose cognitive performance falls significantly below age-matched norms, which may be more clinically relevant for targeting interventions than identifying all patients with MoCA scores < 26 regardless of age.

**Figure 6:**
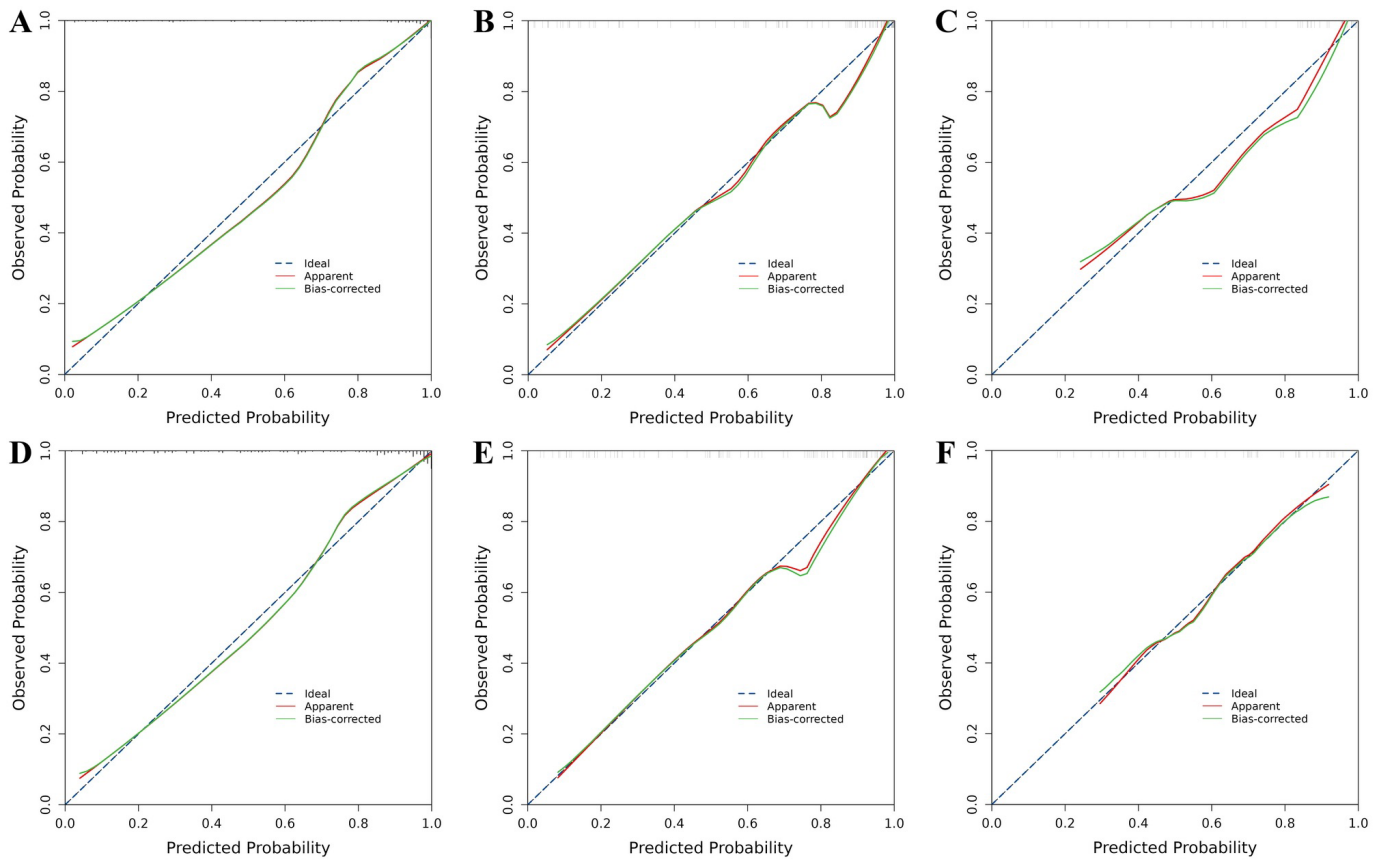
Calibration plots comparing predicted *versus* observed probabilities of PSCI. (A–C) Training, internal validation, and external validation cohorts for the standard-criteria model. (D–F) Training, internal validation, and external validation cohorts for the age-corrected model.

Calibration plots for both the standard-criteria model (Figs. 6A–6C) and age-corrected model (Figs. 6D–6F) demonstrated excellent agreement between predicted and observed PSCI rates across all cohorts. The calibration curves approximated the ideal diagonal in both models, confirming their validity and reliability for risk stratification when applied to the validation datasets.

### Decision curve analysis

**Figure 7:**
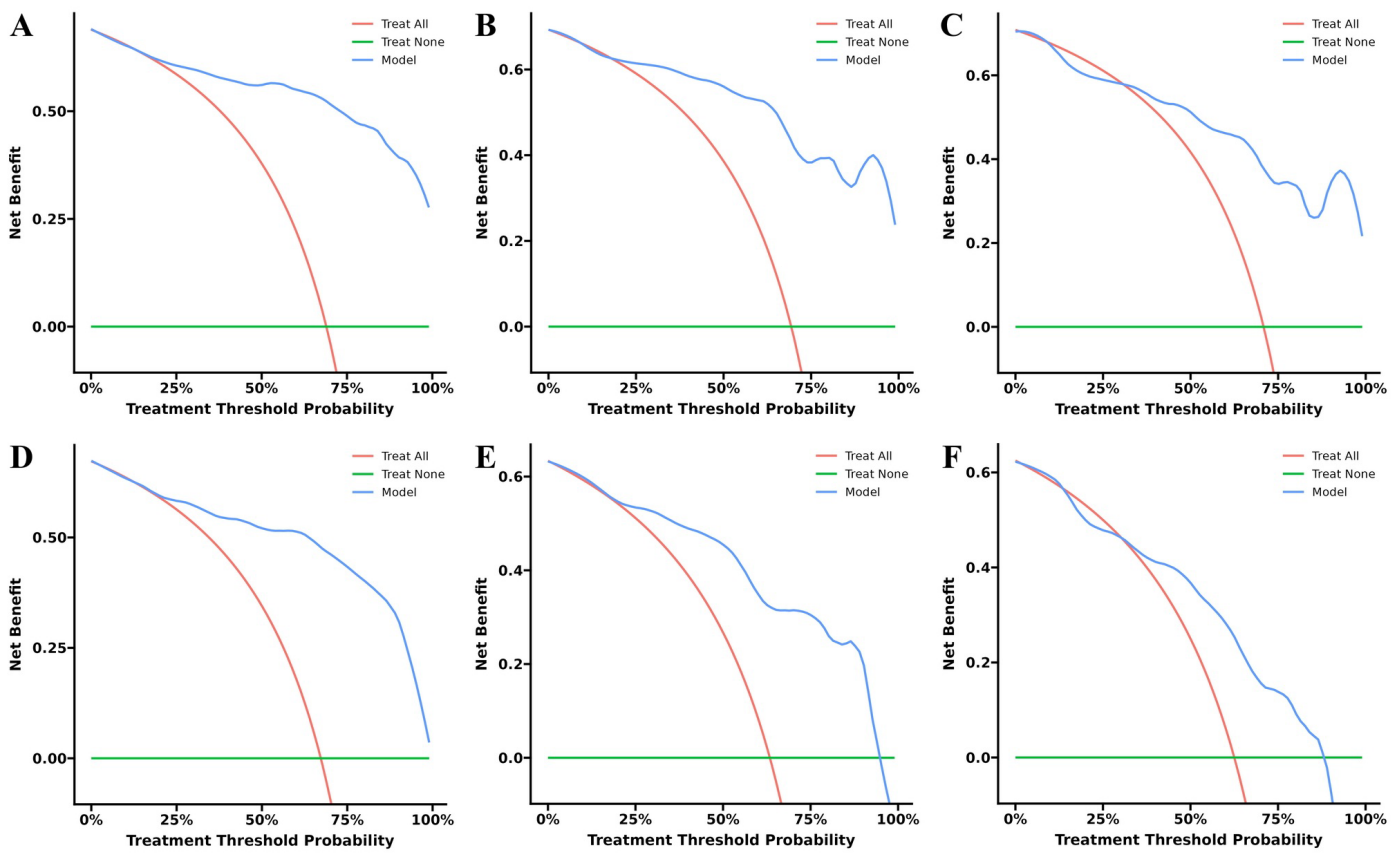
Decision curve analysis demonstrating clinical utility of the nomograms. (A–C) Training, internal validation, and external validation cohorts for the standard-criteria model. (D–F) Training, internal validation, and external validation cohorts for the age-corrected model.

Decision curve analysis revealed that both the standard-criteria model (Figs. 7A–7C) and age-corrected model (Figs. 7D–7F) conferred substantial net clinical benefit across a wide range of threshold probabilities, demonstrating their clinical utility for PSCI risk stratification and supporting their practical application in clinical decision-making.

## Discussion

This study developed and validated two complementary predictive models for PSCI that integrates demographic, clinical, and neuroimaging factors: a standard-criteria model using the conventional MoCA cutoff (<26) and an age-corrected model using age-specific MoCA thresholds. Both models identified the same five independent predictors of PSCI: age, female gender, LDL-C levels, key area cerebral infarction, and a GCA scale score of ≥2. While the two models share identical predictors, they differ in their diagnostic thresholds and clinical applications, offering complementary approaches to PSCI risk stratification. These findings are consistent with existing literature while offering novel insights into the combined predictive value of these factors.

Notably, key area cerebral infarction exhibited the high risk contribution (OR = 6.22 in the standard-criteria model; OR = 5.83 in the age-corrected model), which aligns with the mechanism in brain network theory where damage to critical nodes impairs cognitive compensatory capacity (Brodtmann et al., 2021; Elendu et al., 2023). For instance, thalamic projection fibers connect to the hippocampus and frontoparietal lobes, influencing emotions, executive functions, and learning abilities. The frontal cortex integrates projection signals from other regions, contributing to executive functions and attention, while medial temporal lobe atrophy impairs orientation and recall. The parietal cortex encompasses attention-related areas (Griffin, 2015). Previous research has reported a higher PSCI incidence among patients with infarcts in the left frontotemporal lobes, right parietal lobe, and left thalamus is consistent with our findings (Sun, Tan & Yu, 2014; Zhao et al., 2018; Weaver et al., 2021).

A novel finding of this study is the graded predictive value of the GCA scale. Patients with a GCA score of ≥2 faced an 8.5-fold increased risk of PSCI in the standard-criteria model and a 5.39-fold increased risk in the age-corrected model, while even those with a score of 1 exhibited a 2.79-fold risk trend in the standard model and a 2.02-fold trend in the age-corrected model, providing clinicians with a quantifiable neuroimaging tool for risk assessment. The underlying mechanism may involve the brain white matter, a critical conduit for information transmission, where damage disrupts neural signaling. White matter lesions, a hallmark of cerebral small vessel disease, are strongly associated with reduced executive functions and slower information processing speeds. Furthermore, white matter atrophy, a feature of neurodegenerative processes, leads to reduced brain volume and neuronal loss, impairing memory, executive functions, and reasoning, ultimately contributing to cognitive deficits (Schellekens et al., 2025). These observations are supported by recent diffusion tensor imaging studies, which demonstrate a positive correlation between the extent of white matter fiber integrity disruption and cognitive impairment (Zhang et al., 2025). Furthermore, our findings regarding the GCA scale and key area infarction resonate with the concept of “silent” pathology. Previous research has demonstrated that silent lacunar infarcts are strong predictors of cognitive decline, with elderly individuals harboring such lesions experiencing both increased dementia risk and steeper cognitive deterioration compared to those without these lesions (Vermeer et al., 2003; Blanco-Rojas et al., 2013). The Rotterdam Scan Study found that silent brain infarcts doubled the risk of dementia and were associated with accelerated cognitive decline across multiple domains (Vermeer et al., 2003). While our study utilized visual rating scales, these likely serve as proxies for the accumulated burden of cerebral small vessel disease, capturing both overt infarctions and subclinical vascular pathology that collectively impair cognitive function.

Regarding age, our standard-criteria model confirmed an 18% increase in PSCI risk per additional year (OR = 1.18), while the age-corrected model showed a 13% increase per year (OR = 1.13), reflecting the incorporation of age-adjusted diagnostic thresholds. The risk increment exceeds baseline levels observed in community populations, suggesting that stroke may accelerate age-related cognitive impairment (Yiin et al., 2014; Chau et al., 2023). Female gender emerged as a potent independent risk factor (OR = 4.71 in the standard model; OR = 4.88 in the age-corrected model), aligning with evidence of sex-specific disparities in cognitive outcomes, potentially driven by postmenopausa hormonal shifts (Exalto et al., 2023). This sex disparity is striking, with female patients exhibiting a 4.71-fold higher PSCI risk than males in the standard-criteria model. While some studies argue that sex differences wane after the acute stroke phase (Bako et al., 2022), our data suggest that female gender remains an independent risk factor for long-term cognitive prognosis, possibly due to diminished neuroprotective mechanisms following postmenopausal estrogen impairment (Dong et al., 2020). Additionally, the robust association with LDL-C (OR = 4.50 in the standard model; OR = 3.25 in the age-corrected model) provides new evidence supporting lipid management, as dyslipidemia may contribute to post-stroke vascular cognitive impairment and neurodegeneration through mechanisms such as endothelial dysfunction, blood–brain barrier disruption, and white matter damage (Iadecola et al., 2019; Yang et al., 2022). The attenuated effect size of LDL-C in the age-corrected model (OR 3.25 *vs.* 4.50) suggests that some of the variance attributed to metabolic factors in the standard model may reflect age-related metabolic changes rather than pathological cognitive impairment. The identification of elevated LDL-C as a robust predictor aligns with the “metabolic-cognitive” hypothesis, which posits that metabolic syndrome components induce microvascular endothelial injury and blood–brain barrier disruption. Studies have demonstrated that metabolic dysfunction triggers chronic inflammation and oxidative stress, leading to increased blood–brain barrier permeability, particularly in regions such as the hippocampus, which is critical for cognitive function (Van Dyken & Lacoste, 2018). This microvascular injury contributes to white matter rarefaction and microstructural alterations even before the index stroke occurs, as evidenced by diffusion tensor imaging studies showing reduced fractional anisotropy and increased mean diffusivity in patients with metabolic syndrome (Segura et al., 2010). Furthermore, metabolic syndrome components have been independently associated with silent lacunar infarcts, periventricular white matter hyperintensities, and subcortical white matter lesions, all of which are established contributors to cognitive impairment (Bokura et al., 2008). The vascular damage induced by dyslipidemia may thus represent a continuum of cerebral small vessel disease that begins subclinically and accelerates following acute ischemic events.

Both models demonstrated excellent discriminatory performance across all cohorts. The standard-criteria model achieved AUC values of 0.935 in the training cohort, 0.929 in the internal validation cohort, and 0.884 in the external validation cohort. The age-corrected model showed comparable but lower discriminative capacity, with AUC values of 0.912 in the training cohort, 0.873 in the internal validation cohort, and 0.776 in the external validation cohort. The reduction in AUC for the age-corrected model likely reflects the refined diagnostic approach that accounts for normal age-related cognitive changes, resulting in a more challenging classification task but potentially more clinically meaningful predictions. These results are consistent with earlier PSCI models, which typically reported AUC values of 0.81 to 0.93 (Gong et al., 2021; Dong et al., 2021; Li et al., 2023). The consistent performance between training and validation cohorts in both models indicates that our dual-model approach captures the core pathophysiological mechanisms of PSCI rather than cohort-specific associations. Moreover, predictive accuracy was sustained in the external validation cohort for both models(standard-criteria model AUC = 0.884; age-corrected model AUC = 0.776), demonstrating robust generalizability.

Importantly, classification metrics beyond AUC demonstrated that both models maintain balanced performance in identifying patients with and without PSCI. For the standard-criteria model, precision ranged from 0.88–0.91, recall from 0.91–0.93, specificity from 0.80–0.86, and F1-scores from 0.89–0.92 across cohorts. The age-corrected model showed comparable metrics with precision of 0.86–0.89, recall of 0.89–0.91, specificity of 0.79–0.84, and F1-scores of 0.88–0.90. These balanced metrics across both sensitivity and specificity confirm that neither model simply predicts the majority class, but rather provides meaningful risk discrimination across the full spectrum of PSCI risk.

We developed both a standard-criteria model and an age-corrected model using age-specific cutoffs. The age-corrected model is particularly valuable for clinical practice as it distinguishes pathological cognitive impairment from normal age-related cognitive changes. This distinction is critical for several reasons. First, it helps avoid over-diagnosis in elderly patients, whose lower MoCA scores may reflect normal aging rather than stroke-induced impairment. Second, it prevents under-diagnosis in younger patients, who may score ≥26 yet still demonstrate cognitive decline relative to age-matched peers. Third, by identifying patients whose performance falls below age-expected norms, the age-corrected model better targets those most likely to benefit from intensive cognitive rehabilitation. The standard-criteria model (AUC = 0.935) may be more appropriate for broad screening purposes, identifying all patients with MoCA <26 who may warrant cognitive assessment. In contrast, the age-corrected model (AUC = 0.912) is better suited for precision intervention, focusing resources on patients with cognitive performance substantially worse than age-matched healthy individuals. Clinicians can select between these models based on their specific objectives: universal screening *versus* targeted intervention planning.

The clinical relevance of this study is multifaceted. First, the nomogram derived from both models offer a user-friendly tools for individualized risk assessment, enabling clinicians to efficiently estimate personalized risks. For example, a 60-year-old female patient with LDL-C level of 3.0 mmol/L and key area cerebral infarction has a predicted PSCI probability of 91.1% using the standard-criteria model (as shown in Fig. 4B) and 90.8% using the age-corrected model (Fig. 4D). Such precise risk stratification supports shared decision-making in post-stroke care planning. Second, the identification of key area cerebral infarction and a GCA score of ≥2 as potent predictors underscores the importance of neuroimaging in PSCI risk evaluation. These imaging markers can aid clinicians in pinpointing high-risk patients who may benefit from early cognitive interventions or intensified monitoring. Additionally, the incorporation of readily accessible clinical variables, such as age, sex, and LDL-C levels, enhances both models’ utility in routine clinical practice.

The predictive performance of both models suggests their potential applicability in both clinical and research settings. In clinical practice, the models could serve as complementary screening tools to identify patients at the highest risk for PSCI, thereby enabling targeted interventions such as cognitive rehabilitation or pharmacotherapy. The standard-criteria model may be preferred for initial broad screening to capture all at-risk patients, while the age-corrected model can refine selection for resource-intensive interventions by focusing on those with cognitive performance significantly below age-matched norms. For research, the models could aid in stratifying subjects for clinical trials testing interventions to prevent or mitigate PSCI. The inclusion of modifiable risk factors like LDL-C levels suggests that lipid-modifying interventions could potentially improve cognitive outcomes, although this requires confirmation in prospective studies.

Several limitations merit consideration. First, several important limitations of using the MoCA as our outcome measure warrant discussion. The MoCA has been criticized for being suboptimal in stroke populations due to its heavy reliance on language abilities, which can confound assessment in patients with aphasia, and its underrepresentation of cognitive domains frequently impaired after stroke, such as spatial neglect, apraxia, processing speed, and nonverbal memory. Second, while we addressed this by developing an age-corrected model using published Chinese normative data, this approach has limitations. Our study lacked a healthy control group to establish ideal age-MoCA norms specific to our population. Instead, we relied on published normative data (Lu et al., 2011; Chen et al., 2016), which may not perfectly generalize to our specific cohort. Future studies should incorporate age-matched healthy control groups to establish more precise population-specific norms and validate our age-correction approach. Despite these limitations, the MoCA remains the most widely used screening tool in stroke research and clinical practice globally due to its brevity, ease of administration, and free availability, and our findings provide clinically actionable risk stratification that can guide early intervention decisions.

Third, the cross-sectional design with single time-point assessment at 6 months precludes determination of the causal relationship between risk factors and PSCI and cannot distinguish pre-existing cognitive decline from stroke-induced impairment. Longitudinal cognitive assessment with baseline pre-stroke evaluation or repeated post-stroke measurements would better capture true cognitive change attributable to stroke. Future prospective studies should incorporate serial cognitive assessments to model trajectories of post-stroke cognitive change rather than single time-point cognitive status. Fourth, reliance on visual GCA scale scoring may introduce subjective bias; future studies could enhance objectivity by integrating quantitative measures of white matter hyperintensity volume or automated brain volumetric analysis. Fifth, the comparatively limited sample sizes in specific subgroups may have constrained our statistical power to identify more subtle relationships, particularly for variables showing a trend toward significance (*e.g.*, GCA scale score of 1). Sixth, the selective inclusion criteria (exclusion of patients with severe stroke, significant aphasia, or pre-existing cognitive impairment) limits the generalizability of our findings to the broader stroke population. Both models are most applicable to patients with mild-to-moderate ischemic stroke without severe baseline disability, and external validation in more diverse stroke populations is warranted. Finally, an important area for future investigation is the validation of both nomograms in the “oldest old” population (aged ≥85 years), who present distinct challenges for PSCI risk stratification due to higher prevalence of mixed pathology (concurrent Alzheimer’s disease biomarkers and cerebrovascular disease), making it difficult to attribute cognitive impairment solely to stroke, as well as competing mortality risks, higher baseline rates of pre-existing cognitive impairment, and potentially different risk factor profiles where some traditional vascular risk factors show paradoxical or attenuated associations with outcomes in very advanced age. Our current study included only one patient aged 86 years, limiting our ability to draw conclusions for this subgroup.

Future research should address these shortcomings while building on our findings. Larger, multicenter studies with extended follow-up periods, demographically adjusted cognitive assessments, comprehensive neuropsychological batteries, and serial cognitive measurements could refine both models and uncover additional risk factors while better capturing the dynamic nature of post-stroke cognitive changes. Additionally, head-to-head comparisons in diverse populations and intervention studies using model-based risk stratification could further elucidate the optimal contexts for applying each model in clinical practice.

## Conclusion

This study developed and validated two complementary nomograms for predicting PSCI in acute ischemic stroke patients. Both the standard-criteria and age-corrected model identified five robust predictors: age, female gender, elevated LDL-C, key area cerebral infarction, and GCA scale ≥2. The standard-criteria model achieved excellent discrimination (AUCs 0.884−0.935) and is optimal for broad screening, while the age-corrected model (AUCs 0.776−0.912) provides refined risk stratification by accounting for normal age-related cognitive changes, making it particularly suitable for precision intervention planning. These nomograms offer clinically practical, complementary tools for individualized PSCI risk assessment using readily available variables, enabling early identification and targeted intervention to reduce the substantial socioeconomic burden of PSCI.

##  Supplemental Information

10.7717/peerj.21050/supp-1 Supplemental Information 1 Raw data

## Additional Information and Declarations

### Data Availability

The following information was supplied regarding data availability: The raw data are available in the Supplemental File.

### Clinical Trial Registration

The following information was supplied regarding Clinical Trial registration: 2019-145.
